# Sex Difference in Coronary Artery Spasm Tested by Intracoronary Acetylcholine Provocation Test in Patients with Nonobstructive Coronary Artery Disease

**DOI:** 10.1155/2022/5289776

**Published:** 2022-09-09

**Authors:** Ji Young Park, Se Yeon Choi, Seung-Woon Rha, Byoung Geol Choi, Yung-Kyun Noh, Yong Hoon Kim

**Affiliations:** ^1^Division of Cardiology, Department of Internal Medicine, Nowon Eulji Medical Center, Eulji University, School of Medicine, Seoul, Republic of Korea; ^2^Division of Cardiology, Department of Internal Medicine, Korea University Guro Hospital, Seoul, Republic of Korea; ^3^Department of Computer Science, Hanyang University, Seoul, Republic of Korea; ^4^Division of Cardiology, Department of Internal Medicine, Kangwon National University School of Medicine, Chuncheon, Republic of Korea

## Abstract

**Introduction:**

Cardiovascular diseases manifest differently in men and women. The purpose of this study is to compare the sex difference in the characteristics of coronary artery spasm (CAS) in patients with nonobstructive cardiovascular disease (NOCVD) and the clinical outcomes in accordance with sex in CAS patients.

**Methods:**

The study analysed 5,491 patients with NOCVD who underwent an acetylcholine provocation test from November 2004 to May 2014 for evaluation of chest pain. CAS was defined as greater than 70% of luminal narrowing of the artery during the acetylcholine provocation test.

**Results:**

The patients were divided into men (*n* = 2,506) and women (*n* = 2,985). Mean follow-up days were 1,218 ± 577 days. To adjust for confounding factors, the propensity score matching (PSM) analysis was performed in all patients and among the CAS patients. After PSM analysis, a total of 1,201 pairs in all patients and a total of 713 pairs in CAS patients were generated. In all patients, women showed significantly less incidence of CAS compared with men (62.3% vs 50.9%, *P* < 0.01). Myocardial bridge (MB) and moderate stenosis were less prevalent in women, while transient ST elevation and ischemic chest pain during provocation were more frequent in women. In CAS patients, men had a higher incidence of multivessel spasm than women (35.7% vs. 29.7%, *P* < 0.01). Old age, dyslipidemia, and MB were independent risk factors of CAS in both men and women. In CAS patients, there was no statistical differences for various individual and composite major outcomes up to five years in either men or women. In men with CAS, old age was a risk factor of a 5-year major adverse cardiac event (MACE), and moderate stenosis was a risk factor of both 5-year MACE and 5-year recurrent angina. In women with CAS, mild stenosis was a risk factor of 5-year MACE, while myocardial bridge was a risk factor of 5-year recurrent angina.

**Conclusions:**

In this study, there were sex differences in the angiographic and clinical parameters during the acetylcholine provocation test, incidence of CAS, risk factors of CAS, 5-year MACE, and recurrent angina. Old age, dyslipidemia, and MB were independent risk factors of CAS in both sexes. However, major clinical outcomes up to five years in CAS patients were not different according to sex.

## 1. Introduction

Coronary artery spasm (CAS) is classified as a kind of coronary artery disease (CAD) without obstructive coronary lesions, and it may play an important role in the pathogenesis related to a wide range of cardiac diseases including angina, arrhythmia, acute myocardial infarction (MI), and sudden death [1, 2]. Although the exact pathophysiology of coronary artery spasm is not well known, recent studies have reported that endothelial dysfunction and hypersensitivity of vascular smooth muscle, autonomic tone elevation, oxidative stress, and genetic susceptibility increase contribute to coronary artery spasm [3, 4]. According to a study on the relationship between endothelial function and sex hormones, it was reported that estrogen increases endothelial-dependent vasodilation [5, 6]. In addition, it was reported that there were differences in the prevalence, clinical symptoms, and outcomes of CAD patients according to sex [7–11]. Women have a low-risk profile; therefore, they showed less prevalence of obstructive CAD compared with men [7, 9]. Furthermore, the prevalence and outcomes with no or nonobstructive lesions were different between men and women [8, 10, 11]. There are acetylcholine and ergonovine provocation tests to evaluate the presence of CAS. In our previous studies, we reported that approximately 40% of patients who had typical or atypical symptoms without significant coronary lesions showed positive results in the acetylcholine provocation test [12, 13]. In this study, the propensity score matching (PSM) analysis was performed in all patients and among the CAS patients to adjust confounding factors. After that, we investigated clinical characteristics, angiographic findings according to sex in all patients and CAS patients, and independent risk factors affecting CAS according to sex. We also investigated independent risk factors affecting clinical outcomes and recurrent angina in CAS patients according to sex.

## 2. Materials and Methods

The design of this registry has been introduced before [12, 14, 15]. In brief, it is a single-center, prospective, and all-comer observational registry designed to reflect the “real-world” practice since 2004. Data were collected by trained study coordinators with a standardized case report form. Standardized definitions of all patient-related variables and clinical diagnoses were used. The participants or their legal guardians were given a thorough literal and verbal explanation of the study procedures before granting written consent to participate in the study. The Institutional Review Board (IRB) of Korea University Guro Hospital (KUGH) approved all consenting procedures. The authors of this manuscript have certified that the information contained herein is true and correct as reflected in the records of the IRB (#KUGH10045). KUGH-IRB specifically approved this entire study.

A total of 10,177 patients with typical or atypical chest pain underwent coronary angiography (CAG) at the Cardiovascular Center of KUGH, Seoul, South Korea, between November 2004 and May 2014 were enrolled. Among these, 6,430 patients with typical or atypical chest pain without significant CAD (defined as having a stenosis diameter of less than 70% on quantitative coronary angiography) underwent the intracoronary acetylcholine provocation test. Patients were excluded if they had any of the following conditions: coronary artery bypass graft (CABG), prior percutaneous coronary intervention (PCI), prior cerebrovascular disease (CVD), advanced heart failure (New York Heart Association class III or IV), or serum creatinine ≥2 mg/dl because these conditions could be major causes for adverse cardiovascular events and could bias the results. Finally, a total of 5,491 eligible patients were enrolled.

### 2.1. Study Definitions

Major adverse cardiovascular events (MACE) were defined as the composite of total death, MI, and revascularization including PCI and CABG. Significant CAS was defined as greater than 70% of luminal narrowing of the artery during the acetylcholine provocation test regardless of ischemic electrocardiogram (ECG) changes or the presence of chest pain. Deaths were regarded to be of cardiac cause unless a noncardiac death could be confirmed. Repeated CAG was performed in patients who complained of recurrent angina despite adequate antianginal medication for at least 6 months since the onset of the first CAG. In this case, the physician assumed that CAS may be progressed or there may be newly developing atherosclerotic CAD.

### 2.2. Acetylcholine Provocation Test

The design of the acetylcholine provocation test has been introduced before [12, 14–17]. An initial investigation for CAG included clinical history taking and noninvasive stress tests such as treadmill test, stress echocardiography, and radionuclide studies. And then the CAG was performed to confirm the presence of significant CAD. However, CAG was immediately performed without functional studies in the case of typical resting ischemic chest pain to confirm CAS. Vasodilators such as nitrates, calcium channel blockers (CCB), beta-blockers, nicorandil, and molsidomine were discontinued at least 72 hours before the CAG. CAS induction was tested by intracoronary injection of the acetylcholine immediately after a diagnostic angiography by either a transradial or transfemoral approach. The acetylcholine was injected by incremental doses of 20 (A1), 50 (A2), and 100 (A3) *μ*g/min into the left coronary artery over a 1-minute period with 5-minute intervals up to the maximally tolerated dose under continuous monitoring by ECG and measuring blood pressure. Provocation of the right coronary artery was not performed routinely due to safety issues, as the insertion of a temporary pacemaker is needed to prevent advanced atrioventricular (AV) block during acetylcholine infusion. The angiography was repeated after each acetylcholine dose until a significant focal or diffuse narrowing of greater than 70% was observed by visual assessment. If a significant focal or diffuse vasoconstriction (>70%) of coronary arteries was induced at A1 or A2 dose, additional acetylcholine infusion was stopped for safety because most of the patients underwent the acetylcholine provocation test at the outpatient department base. Intracoronary injection of 0.2 mg of nitroglycerine was administered after completing the acetylcholine provocation test, followed by a CAG 2 minutes later. End-systolic images for each segment of the left coronary artery were chosen according to the corresponding points on the electrocardiographic trace (QRS onset or end of *T* wave) and analysed using the proper quantitative coronary angiography (QCA) system of the catheterization laboratory (FD-20, Phillips, Amsterdam, The Netherlands). The coronary artery diameters were measured by QCA before and after the administration of acetylcholine at the site that showed the greatest changes following drug administration. Reference vessel diameters (RVD) were measured at the proximal and distal portions of each artery. The mean RVD was used to assess diameter narrowing by QCA. Myocardial bridge (MB) was defined as the characteristic phasic systolic compression of the coronary artery with a decrease of more than 30% in diameter on the angiogram after intracoronary nitroglycerin infusion, mostly in anterior-posterior cranial or right anterior oblique cranial projections. Multivessel spasm was defined as significant CAS of more than 2 major epicardial arteries. Diffuse CAS was defined as significant CAS with a side length of more than 20 mm. Spontaneous spasm was defined as focal or diffuse narrowing of greater than 30% of baseline CAG, compared with the RVD after nitroglycerin administration by the intracoronary route.

### 2.3. Study Endpoint

The primary endpoint was the incidence of total death, MI, de novo PCI, and MACE [18]. The secondary endpoint was recurrent angina requiring repeat CAG [12, 14, 15, 19, 20]. In this study, the mean follow-up period was 1,218 ± 577 days (after PSM: 1,235 ± 581) and we could follow up on the clinical data of all enrolled patients through face-to-face interviews at a regular outpatient clinic, medical chart reviews, and telephone contacts.

### 2.4. Statistical Analysis

For continuous variables, differences between the two groups were evaluated by unpaired *t*-test or Mann–Whitney rank test when appropriate. Data were expressed as mean ± standard deviation. For discrete variables, differences were expressed as counts and percentages and analysed with *χ*^2^ or Fisher's exact test between the two groups. To adjust for any potential confounders, propensity score matching (PSM) analysis was performed using the logistic regression model. A total of two PSM populations were generated based on all populations for evaluation of CAS characteristics and only CAS patients were assessed for clinical outcomes according to sex. We tested all available variables that could be of potential relevance: age, cardiovascular risk factors (hypertension, diabetes, dyslipidemia, current smokers, and current alcohol drinkers), angiographic parameters (MB, acetylcholine dose such as 20, 50, and 100 *μ*g/min), CAS site (left anterior descending and left circumflex), CAS length, ECG change, chest pain, AV block, and medical treatment (renin-angiotensin-aldosterone inhibitors, CCBs, nitrate, trimetazidine, molsidomine, beta-blockers, diuretics, aspirin, and statins). Matching was performed with the use of 1 : 1 matching protocol without replacement (nearest neighbour matching algorithm), with a calliper width equal to 0.01 of the standard deviation of the propensity score. Various clinical outcomes were estimated with the Kaplan–Meier method, and differences between the groups were compared with the log-rank test before and after PSM. Cox-proportional hazard models were used to assess the hazard ratio for MACE and recurrent angina according to sex. For all analyses, a two-sided *P* < 0.05 was considered statistically significant. All data were processed with SPSS (version 20.0, SPSS-PC, Inc., Chicago, Illinois).

## 3. Results

In this study, 5,491 patients with NOCVD underwent the acetylcholine provocation test from November 2004 to May 2014 for evaluation of chest pain. The patients were divided into men (*n* = 2,506) and women (*n* = 2,985). Mean follow-up days were 1,218 ± 577 days. To adjust for confounding factors, we utilized the PSM analysis in all patients and among the CAS patients. After PSM analysis, a total of 1,201 pairs in all patients and a total of 713 pairs in CAS patients were generated. In all patients, women showed significantly less incidence of CAS compared with men (62.3% vs 50.9%, *P* < 0.01).

Baseline characteristics of all patients are listed in Table 1. In all patients, women were of older age and had higher left ventricular ejection fractions than men. However, men had higher mean values of blood pressure, body mass index (BMI), and higher rate of smokers and drinkers than women. After PSM analysis, baseline characteristics were similar between women and men. Angiographic findings during the acetylcholine provocation test were listed in Table 1. In all patients, women had less incidence of CAS than men (63% vs 51%, *P* < 0.01). MB and moderate stenosis (50–70%) were less prevalent in women, but transient ST segment elevation and ischemic chest pain during the acetylcholine provocation were more frequent in women.

In CAS patients, baseline characteristics are listed in Table 2. It showed a similar pattern compared with all patients. In discharge medications of CAS patients, men had a higher prescription rate of nitrate and aspirin than women. However, after PSM, women and men had similar baseline characteristics and medications in CAS patients.

In CAS patients, angiographic findings are listed in Table 3. Women had a higher rate of mid to distal spasms than men. However, women had less severe luminal narrowing, a lower prevalence of multivessel spasm, left circumflex artery spasm, and proximal to distal spasm than men. After adjusting confounding factors using PSM, the angiographic findings were balanced between women and men. However, during the acetylcholine provocation test, women had a smaller reference diameter and a larger minimum narrowing diameter than men. 

Clinical outcomes up to five years are listed in Table 4. There was a trend toward a higher incidence of de novo PCI in men. However, there was no statistical significance, and there were no statistical difference for various individual and composite major outcomes up to five years in both men and women.

**Figure 1:**
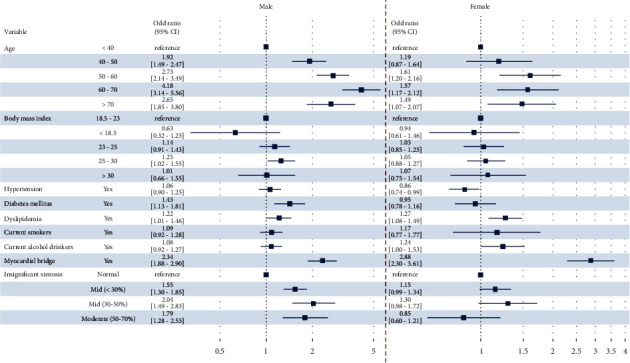
Forest plot of odds ratio and 95% confidence intervals for acetylcholine-induced coronary artery spasm by univariate logistic regression analysis. CI, confidence interval.

We investigated independent risk factors for CAS in men and women using a multivariate Cox-proportional hazard ratio model. In both men and women, old age, dyslipidemia, and MB were independent risk factors of CAS. However, diabetes mellitus and insignificant stenosis in men and alcohol drinkers in women were associated with a higher incidence of CAS. Hypertension or uncontrolled blood pressure were associated with a lower incidence of CAS in women (Figure 1).

We investigated independent risk factors for 5-year MACE in CAS patients using a multivariate Cox-proportional hazard ratio model. In men with CAS, old age (hazard ratio (HR): 1.09; 95% confidence interval (CI): 1.02–1.17) and moderate stenosis (50–70%, HR: 6.15; CI: 1.12–33.5) were independent risk factors of 5-year MACE. In women with CAS, insignificant stenosis (30–50%, HR: 18.1; CI: 1.14–287.3) was an independent risk factor of 5-year MACE (Table 5).

We also investigated independent risk factors for 5-year recurrent angina in CAS patients using a multivariate Cox-proportional hazard ratio model. In men with CAS, moderate stenosis (50–70%, HR: 2.83; CI: 1.48–5.41) and use of chronic nitrates (HR: 2.35; CI: 1.33–4.16) were independent risk factors of 5-year recurrent angina. In women with CAS, MB (HR: 1.70; CI: 1.04–2.78) and use of chronic nitrates (HR: 1.90; CI: 1.07–3.37) were independent risk factors of 5-year recurrent angina (Table 6).

## 4. Discussion

We investigated the sex differences in the characteristics of CAS in patients with NOCVD and the clinical outcomes according to sex in CAS patients. CAS was assessed using the acetylcholine provocation test.

The main findings of this study were as follows: (1) women had a lower incidence of CAS than men; (2) MB and moderate stenosis (50-70%) were less prevalent in women; (3) Transient ST elevation and ischemic chest pain during provocation were more frequent in women than in men; (4) Age, dyslipidaemia, and MB were independent risk factors for CAS in both men and women, but there were sex differences in some risk factors; diabetes mellitus and insignificant stenosis in men and alcohol drinkers in women were independent risk factorsof the acetylcholine induced CAS; and hypertension or uncontrolled blood pressure were associated with a lower incidence of acetylcholine induced CAS in women; (5) major clinical outcomes up to five years were similar between men and women; and(6) in men with CAS, old age was a risk factor of 5-year MACE, and moderate stenosis was a risk factors of both 5-year MACE and 5-year recurrent angina. In women with CAS, mild stenosis was a risk factor of 5-year MACE, and MB was a risk factor of 5-year recurrent angina.

Among patients suffering from typical or atypical chest pain but no or nonobstructive coronary stenosis, the present study demonstrated more frequent CAS in men (63% vs 51%, *P* < 0.01). It is crucial to know why men had a higher incidence of CAS in this study. An anatomical variant, such as MB, is a suspected factor for this difference in prevalence. MB is an anomaly in which the myocardium overlies some of the coronary artery segments during systole, which was more common in men [21], and it is well known as one of the risk factors inducing CAS [17, 22]. The results of our present study showed that MB had a higher prevalence in men not only in all patients but also in the matched population. For this reason, we suggest that MB is an important factor that explains the difference in the prevalence of CAS according to sex. Despite the lower prevalence of CAS, women had a higher incidence of transient ST elevation and chest pain during the acetylcholine provocation test. This result may be caused by the higher incidence of microvascular angina in women. Approximately 25–35% of patients suspected of CAS are concluded as having microvascular angina instead of epicardial spasm [13, 23, 24]. Therefore, physicians should consider microvascular angina on a negative provocation test, especially for women. Among the male CAS patients, the most prominent feature of angiographic findings in accordance with acetylcholine provocation test was a higher incidence of multivessel spasm. Previous studies exhibited that alcohol drinking was associated with increased multivessel spasm. However, being men was not established to be as an independent risk factor of multivessel spasm [20]. In our study, after adjusting confounding factors using PSM, there was no difference in multivessel spasm incidence in men and women [25].

We used logistic regression analysis to determine which factors are associated with CAS in men and women. The present study showed that age, dyslipidemia, and MB were risk factors affecting CAS in both men and women. However, there were different risk factors between men and women such as diabetes and insignificant stenosis in men, and alcohol drinking and hypertension in women. Diabetes was associated with CAS development in men. It is a discrepancy compared with our previous study which reported that diabetes and blood sugar control were not associated with CAS [26]. However, previous studies did not assess according to sex, and another previous study reported that diabetes was a risk factor for CAS development in men with low hs-CRP [27]. In this study, insignificant stenosis was an independent risk factor of CAS and 5-year MACE in men and women, and was an independent risk factor of 5-year recurrent angina in men. Traditionally, there are different factors to protect against endothelial dysfunction or vasoconstriction between men and women. Oestrogen is well known to have protective effects such as an enhancement of endothelium-dependent relaxation, and even in postmenopausal women. Oestrogen has been reported to contribute to prevent the acetylcholine-induced vasoconstriction in nonobstructive coronary artery disease patients in women; but not in men [28]. Therefore, it might result in less prevalence of CAS in women compared with men. However, further studies are needed to determine the association between hormonal effects and CAS. In this study, hypertension showed a negative effect on CAS development, especially in women. Previous studies suggested some mechanisms and theories as to why hypertension was negatively associated with CAS, including the difference in vascular smooth muscle cell type [27], less effectiveness of the acetylcholine provocation signifying endothelial dysfunction, and the difference in pathology between CAD and CAS [16]. However, further studies, such as a prospective study, are required to specify a definitive conclusion for the sex difference.

In the present study, all CAS patients were prescribed adequate antianginal medications including diltiazem, nitrate, nicorandil, molsidomine, and others depending on the physician's discretion. Individual and composite clinical outcomes up to five years were not different between men and women. It is well known that CAS has a good prognosis for long-term outcomes, but recurrent chest pain leading to repeated angiography should be considered [3, 15]. Our results showed that men had a higher statistical trend for recurrent angina compared with women. However, after PSM analysis, this statistical trend disappeared. Therefore, we assumed that CAS management through adequate medical therapy may play an important role in achieving a good prognosis for both men and women. This result is consistent compared with a previous study showing no sex difference for clinical outcomes [29]. However, independent risk factor for recurrent chest pain were moderate stenosis in men (50–70%, HR: 18.1; CI: 1.14–287.3) , dyslipidemia (HR: 1.67; CI: 0.99–2.82), and MB (HR: 1.70; CI: 1.04–2.78) in women. Therefore, we suggest that CAS treatment should be considered to reduce the incidence of recurrent angina in both men and women with CAS.

There are several limitations to this study. First, it is a retrospective observational study, and selection bias and recall bias may confound the results. Although we adjusted baseline confounding factors related to clinical outcomes using PSM analysis, the factors could not be perfectly adjusted due to underlying limitations. Second, we investigated the CAS using the acetylcholine provocation test, rather than the ergonovine provocation test. Therefore, in the future, it seems necessary to investigate the sex difference of CAS using the ergonovine provocation test [30]. Third, we used a less strict criterium of 70% or more luminal narrowing during the acetylcholine provocation test. For patient safety, the radial approach to the acetylcholine provocation test was performed in an outpatient procedure using the 4Fr radial sheath, rather than at RCA. Furthermore, our provocation protocol was set up before the year 2004; therefore, there are some differences compared with the current protocol and diagnostic criteria [31]. Fourth, in this study, no physiological evaluation was performed on the angiographically insignificant lesions. However, those lesions might develop significantly. In fact, in this study, insignificant lesions were associated with an increased incidence of CAS in men and an independent risk factor of MACE in five years follow-up. Fifth, in this study, we have no data regarding the atrial fibrillation rate or complication rates during the acetylcholine provocation test between men and women. Finally, we did not have detailed medication data before the acetylcholine provocation test or follow-up periods, so we could not evaluate the detailed medication effects on all patients.

## 5. Conclusion

NOCVD is a common disease in CVD patients and is related to CAS, so the acetylcholine provocation test is beneficial for evaluating CAS. In this study, we investigated clinical characteristics and angiographic findings according to sex in all patients and in CAS patients, as well as the independent risk factors affecting CAS according to sex. We also investigated independent risk factors affecting clinical outcomes and recurrent angina in CAS patients according to sex. We found sex differences in the angiographic and clinical parameters during the acetylcholine provocation test, incidence of CAS, risk factors of CAS, 5-year MACE, and 5-year recurrent angina. Old age, dyslipidemia, and MB were independent risk factors of CAS in both sexes. However, major clinical outcomes up to five years in CAS patients were no different according to sex. Therefore, we assumed that sex-specific considerations would be important in CAS diagnosis, and optimal medical therapy for CAS would be considered to prevent recurrent angina requiring follow-up CAG and is helpful for achieving a good prognosis in both men and women.

## Figures and Tables

**Table 1:** Baseline clinical and angiographic parameters in patients who underwent acetylcholine provocation test.

Variables, *N* (%)	Overall patients				Matched patients			
	Men (*n* = 2506)	Women (*n* = 2985)	*P* value	S.diff	Men (*n* = 1201)	Women (*n* = 1201)	*P* value	S.diff
Baseline clinical characteristics								
Age, years	52.4 ± 12.6	57.3 ± 11.7	<0.01	−0.40	54.6 ± 13.3	54.7 ± 12.1	0.966	−0.01
Blood pressure (BP)								
Systolic BP	134 ± 19	136 ± 22	0.019	−0.08	134 ± 20	134 ± 21	0.163	0.03
Diastolic BP	80 ± 12	76 ± 13	<0.01	0.32	79 ± 12	79 ± 13	0.486	−0.01
Heart rate	70 ± 12	72 ± 13	<0.01	−0.15	71 ± 12	71 ± 12	0.975	0.01
Body mass index	24.5 ± 3.0	24.1 ± 3.3	<0.01	0.15	24.3 ± 2.9	24.2 ± 3.5	0.184	0.03
LV ejection fraction (%)	59.0 ± 3.8	59.2 ± 3.7	0.029	−0.05	59.1 ± 3.9	59.3 ± 3.0	0.858	−0.06

Risk factors								
Hypertension	1080 (43.1)	1312 (44.0)	0.524	0.13	509 (42.4)	520 (43.3)	0.650	0.14
Diabetes	396 (15.8)	446 (14.9)	0.378	−0.22	176 (14.7)	185 (15.4)	0.607	0.19
New-onset diabetes	80 (3.2)	118 (4.0)	0.132	0.40	36 (3.0)	40 (3.3)	0.641	0.19
Insulin	41 (1.6)	45 (1.5)	0.702	−0.10	16 (1.3)	21 (1.7)	0.407	0.34
Dyslipidemia	707 (28.2)	829 (27.8)	0.717	−0.08	315 (26.2)	317 (26.4)	0.926	0.03
Current smokers	1040 (41.5)	94 (3.1)	<0.01	−8.13	98 (8.2)	94 (7.8)	0.763	−0.12
Current alcohol drinkers	1384 (55.2)	408 (13.7)	<0.01	−7.10	386 (32.1)	369 (30.7)	0.455	−0.25

Angiographic and clinical parameters during acetylcholine provocation test								
CAS positive	1575 (62.8)	1535 (51.4)	<0.01	−1.52	748 (62.3)	611 (50.9)	<0.01	−1.52
Spontaneous spasm	506 (20.2)	594 (19.9)	0.788	−0.07	254 (21.1)	249 (20.7)	0.802	−0.09
Myocardial bridge	556 (22.2)	434 (14.5)	<0.01	−1.79	273 (22.7)	168 (14.0)	<0.01	−2.04
EKG change	106 (4.2)	124 (4.2)	0.889	−0.04	45 (3.7)	56 (4.7)	0.263	0.45
ST-segment elevation	28 (1.1)	43 (1.4)	0.291	0.29	10 (0.8)	25 (2.1)	0.011	1.03
ST-segment depression	40 (1.6)	39 (1.3)	0.369	−0.24	16 (1.3)	14 (1.2)	0.713	−0.15
T-inversion	19 (0.8)	27 (0.9)	0.553	0.16	8 (0.7)	12 (1.0)	0.369	0.37
Atrial fibrillation	21 (0.8)	22 (0.7)	0.672	−0.11	12 (1.0)	7 (0.6)	0.249	−0.47
Chest pain	1056 (42.1)	1413 (47.3)	<0.01	0.78	485 (40.4)	573 (47.7)	<0.01	1.11
AV block	645 (25.7)	820 (27.5)	0.148	0.34	304 (25.3)	323 (26.9)	0.377	0.31
Insignificant stenosis								
Mild (<30%)	1197 (47.8)	1461 (48.9)	0.384	0.17	567 (47.2)	581 (48.4)	0.567	0.17
Mild (30–50%)	223 (8.9)	231 (7.7)	0.120	−0.40	97 (8.1)	98 (8.2)	0.940	0.03
Moderate (50–70%)	184 (7.3)	139 (4.7)	<0.01	−1.10	71 (5.9)	71 (5.9)	>0.99	0.00

Data are presented as *N* (%) or mean ± standard deviation; SD, standardized difference; LV, left ventricular; CAS, coronary artery spasm; ACh acetylcholine; AV, atrioventricular.

**Table 2:** Baseline clinical, medical treatment, and angiographic characteristics in patients with coronary artery spasms.

Variables, *N* (%)	Overall patients				Matched patients			
	Men (*n* = 1575)	Women (*n* = 1535)	*P* value	S.diff	Men (*n* = 713)	Women (*n* = 713)	*P* value	S.diff
Baseline characteristics								
Age	54.4 ± 11.5	58 ± 11.1	<0.01	−0.31	56.5 ± 12.2	56.4 ± 11.5	0.793	0.01
Blood pressure (BP)								
Systolic BP	133 ± 18	134 ± 21	0.499	−0.02	133 ± 18	133 ± 21	0.537	0.03
Diastolic BP	80 ± 11	75 ± 13	<0.01	0.37	78 ± 11	78 ± 13	0.983	0.00
Heart rate	69 ± 12	71 ± 12	0.002	−0.11	70 ± 12	69 ± 12	0.785	0.01
Pulse pressure	53 ± 14	58 ± 16	<0.01	−0.31	55 ± 15	54 ± 16	0.443	0.04
Body mass index (kg/m^2^)	24.6 ± 2.9	24.1 ± 3.3	<0.01	0.15	24.6 ± 2.9	24.3 ± 3.4	0.146	0.08
LV ejection fraction (%)	59.1 ± 3.3	59.2 ± 3.5	0.575	−0.03	59 ± 3.6	59 ± 3.8	0.877	0.01

Factors of risk								
Hypertension	688 (43.7)	648 (42.2)	0.408	−0.22	296 (41.5)	296 (41.5)	>0.99	0.00
Diabetes	276 (17.5)	225 (14.7)	0.030	−0.72	115 (16.1)	116 (16.3)	0.943	0.03
New-onset diabetes	53 (3.4)	71 (4.6)	0.072	0.63	23 (3.2)	27 (3.8)	0.565	0.30
Insulin	29 (1.8)	20 (1.3)	0.228	−0.43	11 (1.5)	8 (1.1)	0.488	−0.36
Dyslipidemia	468 (29.7)	462 (30.1)	0.815	0.07	200 (28.1)	199 (27.9)	0.953	−0.03
Current smokers	666 (42.3)	52 (3.4)	<0.014	−8.16	60 (8.4)	52 (7.3)	0.431	−0.40
Current alcohol drinkers	882 (56.0)	229 (14.9)	<0.014	−6.92	218 (30.6)	206 (28.9)	0.487	−0.31

Baseline angiographic characteristics								
Myocardial bridge	428 (27.2)	315 (20.5)	<0.014	−1.36	179 (25.1)	178 (25.0)	0.951	−0.03
Insignificant stenosis								
Mild (<30%)	788 (50.0)	773 (50.4)	0.856	0.05	365 (51.2)	358 (50.2)	0.711	−0.14
Mild (30–50%)	160 (10.2)	129 (8.4)	0.092	−0.58	67 (9.4)	66 (9.3)	0.927	−0.05
Moderate (50–70%)	127 (8.1)	63 (4.1)	<0.014	−1.61	43 (6.0)	40 (5.6)	0.734	−0.17

Medications								
Calcium channel blockers	1343 (85.3)	1325 (86.3)	0.402	0.11	610 (85.6)	608 (85.3)	0.881	−0.03
Diltiazem	1307 (83.0)	1285 (83.7)	0.585	0.08	593 (83.2)	595 (83.5)	0.887	0.03
Nitrate	1059 (67.2)	951 (62.0)	0.002	−0.66	461 (64.7)	465 (65.2)	0.824	0.07
Trimetazidine	837 (53.1)	829 (54.0)	0.629	0.12	383 (53.7)	372 (52.2)	0.559	−0.21
Nicorandil	515 (32.7)	463 (30.2)	0.128	−0.45	222 (31.1)	228 (32.0)	0.732	0.15
Molsidomine	122 (7.7)	110 (7.2)	0.538	−0.21	52 (7.3)	41 (5.8)	0.238	−0.60
Beta-blockers	98 (6.2)	122 (7.9)	0.061	0.65	50 (7.0)	54 (7.6)	0.684	0.21
ARBs	243 (15.4)	223 (14.5)	0.482	−0.23	97 (13.6)	109 (15.3)	0.366	0.44
ACE inhibitors	63 (4.0)	41 (2.7)	0.039	−0.73	29 (4.1)	29 (4.1)	>0.99	0.00
Aspirin	222 (14.1)	156 (10.2)	0.001	−1.13	96 (13.5)	96 (13.5)	>0.99	0.00
Statins	606 (38.5)	582 (37.9)	0.748	−0.09	268 (37.6)	269 (37.7)	0.956	0.02

Data are presented as *N* (%) or mean ± standard deviation. SD, standardized difference; LV, left ventricular; ARB, angiotensin receptor blocker; ACE inhibitors, angiotensin-converting enzyme inhibitors.

**Table 3:** Angiographic characteristics in patients with acetylcholine-induced coronary artery spasm.

Variables, *N* (%)	Overall patients				Matched patients			
	Male (*n* = 1575)	Female (*n* = 1535)	*P* value	S.diff	Male (*n* = 748)	Female (*n* = 611)	*P* value	S.diff
Quantitative coronary angiography; QCA								
MND (mm) (during ACh test)	0.67 ± 0.35	0.73 ± 0.35	<0.01	−0.17	0.68 ± 0.36	0.74 ± 0.35	0.002	−0.17
MND (%) (during ACh test)	72.0 ± 12.0	68.0 ± 12.0	<0.01	0.31	72.1 ± 12.5	68.3 ± 13.0	<0.01	0.30
RD (mm) (after NTG injection)	2.44 ± 0.55	2.35 ± 0.83	<0.01	0.14	2.44 ± 0.53	2.37 ± 0.58	0.019	0.11

Acetylcholine dose								
A1 (20 ug/min)	90 (5.7)	73 (4.8)	0.230	−0.42	42 (5.6)	43 (7.0)	0.281	0.57
A2 (50 ug/min)	561 (35.6)	523 (34.1)	0.365	−0.26	265 (35.4)	206 (33.7)	0.509	−0.29
A3 (100 ug/min)	924 (58.7)	939 (61.2)	0.154	0.32	441 (59.0)	362 (59.2)	0.914	0.04

Multivessel spasm	562 (35.7)	456 (29.7)	<0.01	−1.05	255 (34.1)	194 (31.8)	0.362	−0.41

Spasm site								
Left anterior descending	1473 (93.5)	1447 (94.3)	0.387	0.08	698 (93.3)	578 (94.6)	0.326	0.13
Left circumflex	654 (41.5)	522 (34.0)	<0.01	−1.23	299 (40.0)	218 (35.7)	0.105	−0.70

Diffuse spasm								
(Narrowing length >20 mm)	1352 (85.8)	1317 (85.8)	0.972	0.00	640 (85.6)	531 (86.9)	0.475	0.15

Spasm location								
Prox to distal	679 (43.1)	601 (39.2)	0.025	−0.62	302 (40.4)	261 (42.7)	0.383	0.36
Mid to distal	569 (36.1)	626 (40.8)	0.008	0.75	276 (36.9)	229 (37.5)	0.825	0.10
Proximal only	115 (7.3)	117 (7.6)	0.734	0.12	54 (7.2)	49 (8.0)	0.579	0.29
Mid only	181 (11.5)	167 (10.9)	0.588	−0.18	102 (13.6)	66 (10.8)	0.114	−0.81
Distal only	31 (2.0)	24 (1.6)	0.392	−0.30	14 (1.9)	6 (1.0)	0.175	−0.74

Data are presented as *N* (%) or mean ± standard deviation; S.diff, standardized difference; MND, minimum narrowing diameter; ACh, acetylcholine; RD, reference diameter; NTG, nitroglycerin.

**Table 4:** Various clinical outcomes of coronary artery spasm up to 5-year by Kaplan–Meier curve analysis.

Outcomes, incidence (%)	Overall patients			Matched patients		
	Male (*n* = 1575)	Female (*n* = 1535)	Log rank	Male (*n* = 713)	Female (*n* = 713)	Log rank
MACE	13 (1.2)	7 (0.6)	0.201	4 (0.8)	3 (0.6)	0.688
Total death	5 (0.4)	3 (0.3)	0.502	1 (0.1)	2 (0.4)	0.581
Cardiac death	2 (0.1)	1 (0.0)	0.578	0 (0.0)	1 (0.1)	0.322
Myocardial infarction; MI	4 (0.2)	2 (0.1)	0.432	1 (0.2)	0 (0.0)	0.316
CAS-induced MI	4 (0.2)	1 (0.0)	0.187	0 (0.0)	0 (0.0)	—
De novo PCI	5 (0.6)	2 (0.1)	0.278	3 (0.7)	0 (0.0)	0.081
Recurrent angina	104 (10.7)	77 (7.8)	0.059	41 (9.4)	29 (5.3)	0.127

MACE, major adverse cardiac events; CAS, coronary artery spasm; PCI, percutaneous coronary intervention.

**Table 5:** Independent risk factors of MACE in CAS patients at follow-up period by multivariable Cox-proportional hazard ratio model analysis.

Variables	Men		Women	
	Hazard ratio [95% CI]	*P* value	Hazard ratio [95% CI]	*P* value
Age	1.09 [1.02–1.17]	0.006	0.98 [0.90–1.08]	0.767
Hypertension	0.96 [0.29–3.11]	0.953	2.24 [0.35–14.0]	0.388
Uncontrolled blood pressure	0.89 [0.27–2.87]	0.847	1.20 [0.21–6.84]	0.837
Diabetes mellitus	1.00 [0.25–4.00]	0.997	1.22 [0.10–14.3]	0.873
Dyslipidemia	1.65 [0.49–5.55]	0.417	1.09 [0.15–7.75]	0.931
Current smokers	2.18 [0.65–7.23]	0.201	7.77 [0.52–115.4]	0.136
Current alcohol drinkers	0.80 [0.24–2.59]	0.710	1.11 [0.10–11.7]	0.927
Myocardial bridge	2.15 [0.65–7.12]	0.209	0.47 [0.04–5.03]	0.533

Insignificant stenosis				
Mild (<30%)	1.08 [0.24–4.79]	0.919	1.73 [0.17–16.9]	0.636
Mild (30–50%)	0.93 [0.08–9.74]	0.954	18.1 [1.14–287.3]	0.040
Moderate (50–70%)	6.15 [1.12–33.5]	0.036	—	—

Medications				
Calcium channel blockers	0.79 [0.15–3.97]	0.781	—	0.988
Nitrates	1.40 [0.34–5.77]	0.634	3.35 [0.34–32.6]	0.297
Beta-blockers	0.77 [0.08–6.71]	0.813	0.65 [0.03–12.5]	0.776
ARBs	0.13 [0.01–1.73]	0.125	—	0.976
ACE inhibitors	1.03 [0.1–10.04]	0.977	—	0.994
Aspirin	0.98 [0.22–4.33]	0.982	—	0.987
Statins	1.46 [0.39–5.43]	0.563	1.10 [0.14–8.50]	0.927

CAS, coronary artery spasm; CI, confidence interval; ARB, angiotensin receptor blocker; ACE inhibitors, angiotensin-converting enzyme inhibitors.

**Table 6:** Independent risk factors of recurrent angina in CAS patients at follow-up period using multivariable Cox-proportional hazard ratio model analysis.

Variables	Men		Women	
	Hazard ratio [95% CI]	*P* value	Hazard ratio [95% CI]	*P* value
Age	1.01 [0.99–1.03]	0.232	1.00 [0.98–1.02]	0.728
Hypertension	1.01 [0.66–1.54]	0.958	1.07 [0.64–1.79]	0.788
Uncontrolled blood pressure	1.02 [0.68–1.54]	0.888	0.82 [0.51–1.33]	0.445
Diabetes mellitus	0.80 [0.47–1.36]	0.420	0.76 [0.39–1.49]	0.433
Dyslipidemia	1.07 [0.67–1.71]	0.749	1.67 [0.99–2.82]	0.053
Current smokers	1.29 [0.86–1.95]	0.212	1.30 [0.39–4.28]	0.658
Current alcohol drinkers	0.70 [0.46–1.06]	0.093	0.57 [0.25–1.27]	0.174
Myocardial bridge	0.72 [0.44–1.17]	0.192	1.70 [1.04–2.78]	0.034

Insignificant stenosis				
Mild (<30%)	0.93 [0.58–1.49]	0.770	0.73 [0.43–1.25]	0.263
Mild (30–50%)	1.06 [0.49–2.28]	0.867	1.43 [0.66–3.09]	0.353
Moderate (50–70%)	2.83 [1.48–5.41]	0.002	1.72 [0.63–4.72]	0.286

Medications				
Calcium channel blockers	1.72 [0.78–3.77]	0.174	0.73 [0.36–1.47]	0.392
Nitrates	2.35 [1.33–4.16]	0.003	1.90 [1.07–3.37]	0.028
Beta-blockers	1.38 [0.68–2.81]	0.365	1.71 [0.88–3.34]	0.113
ARBs	0.60 [0.31–1.15]	0.129	0.51 [0.23–1.12]	0.097
ACE inhibitors	1.09 [0.45–2.61]	0.844	0.32 [0.04–2.38]	0.267
Aspirin	1.34 [0.80–2.27]	0.262	0.88 [0.43–1.83]	0.749
Statins	1.30 [0.82–2.05]	0.254	1.34 [0.77–2.31]	0.289

CAS, coronary artery spasm; CI, confidence interval; ARB, angiotensin receptor blocker; ACE inhibitors, angiotensin-converting enzyme inhibitors.

## Data Availability

All relevant data can be assessed from the website via the following URL: http://www.ciri.or.kr.
